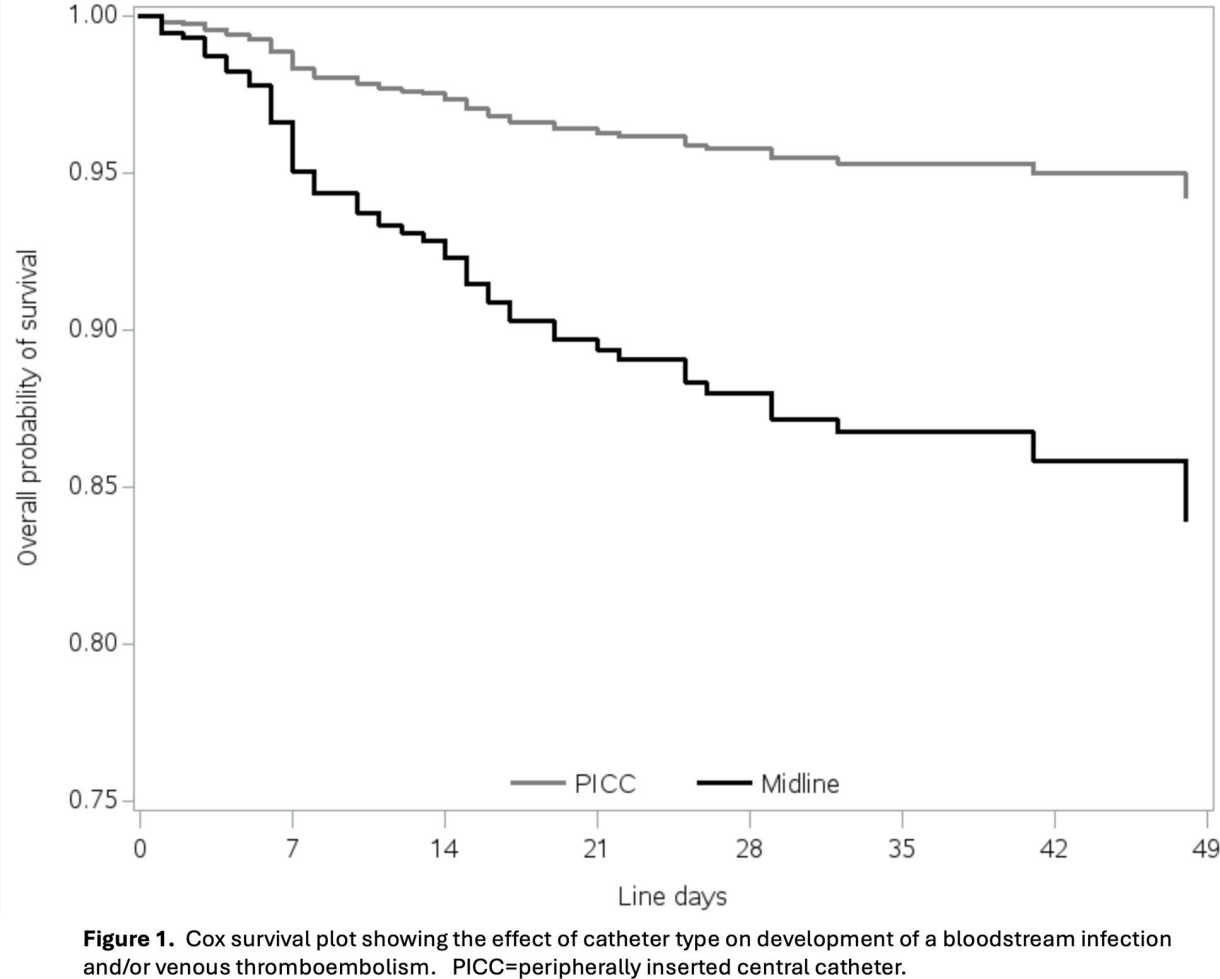# 18 Enhanced C. auris screening and rapid diagnostics in cluster units: association with fewer clinical infections

**DOI:** 10.1017/ash.2026.10467

**Published:** 2026-06-23

**Authors:** Justin Kim, Guadalupe Ordaz-Nielsen, James Vu, Kim Tran, Ho Kim, Michael Schoeny, Michael Lin

**Affiliations:** 1 Rush University Medical Center; 2 UC Davis Medical Center; 3 Rush University

## Abstract

**Background:** The safety of midlines (MLs) versus peripherally inserted central catheters (PICCs) in the context of outpatient parenteral antimicrobial therapy (OPAT) is still debated. The Infectious Diseases Society of America guidelines currently offer a weak recommendation to consider MLs for antibiotic durations of <14 days. The purpose of this study was to quantify the ML complication rate compared to PICCs for OPAT. **Methods:** We performed a single-center, retrospective cohort study of MLs and PICCs placed by the bedside vascular access team for OPAT as recommended by the infectious diseases consult service at a 670-bed urban academic medical center. Between July 2019 and June 2022, 1351 catheters were placed in 1252 unique patients for OPAT. The primary exposure was the placement of a ML versus PICC, informed by selection guidelines from the Infusion Nurses Society. The primary outcome was any bloodstream infection (BSI) or superficial or deep venous thromboembolism (VTE) while the catheter was present. We estimated the hazard ratio (HR) of developing the outcome among MLs compared to PICCs using Cox proportional hazards models, accounting for multiple episodes of OPAT-related catheter placement over time in the same patient and adjusting for confounding by planned antibiotic duration of ≥14 days and the use of vancomycin. We tested whether planned antibiotic duration modified the association between catheter type and the outcome using an interaction term. Results Demographic and clinical characteristics are in Table 1. The ML complication rate was 6.1 per 1000 line-days (26 [3 BSI, 23 VTE] of 322 MLs) versus 1.2 per 1000 line-days for PICCs (40 [11 BSI, 30 VTE] of 1029 PICCs), with a higher complication rate for MLs (adjusted HR [aHR] 2.97, 95% confidence interval [CI] 1.32-6.70, p=0.01). Adjusted survival curves are given in Figure 1. The aHR was 0.97 (95% CI 0.30-3.11, p=0.96) among catheters with a planned antibiotic duration of ≤14 days and 4.11 (95% CI 2.08-8.16, p<0.01) for <14 days (p-interaction=0.03). Conclusion In this study of patients receiving OPAT, complications were uncommon but significantly more frequent for MLs than PICCs. This association appeared to differ by planned antibiotic durations of <14 days than ≤14 days. Our study calls into question the safety of MLs for OPAT, especially for prolonged durations of therapy.

**Figure:**
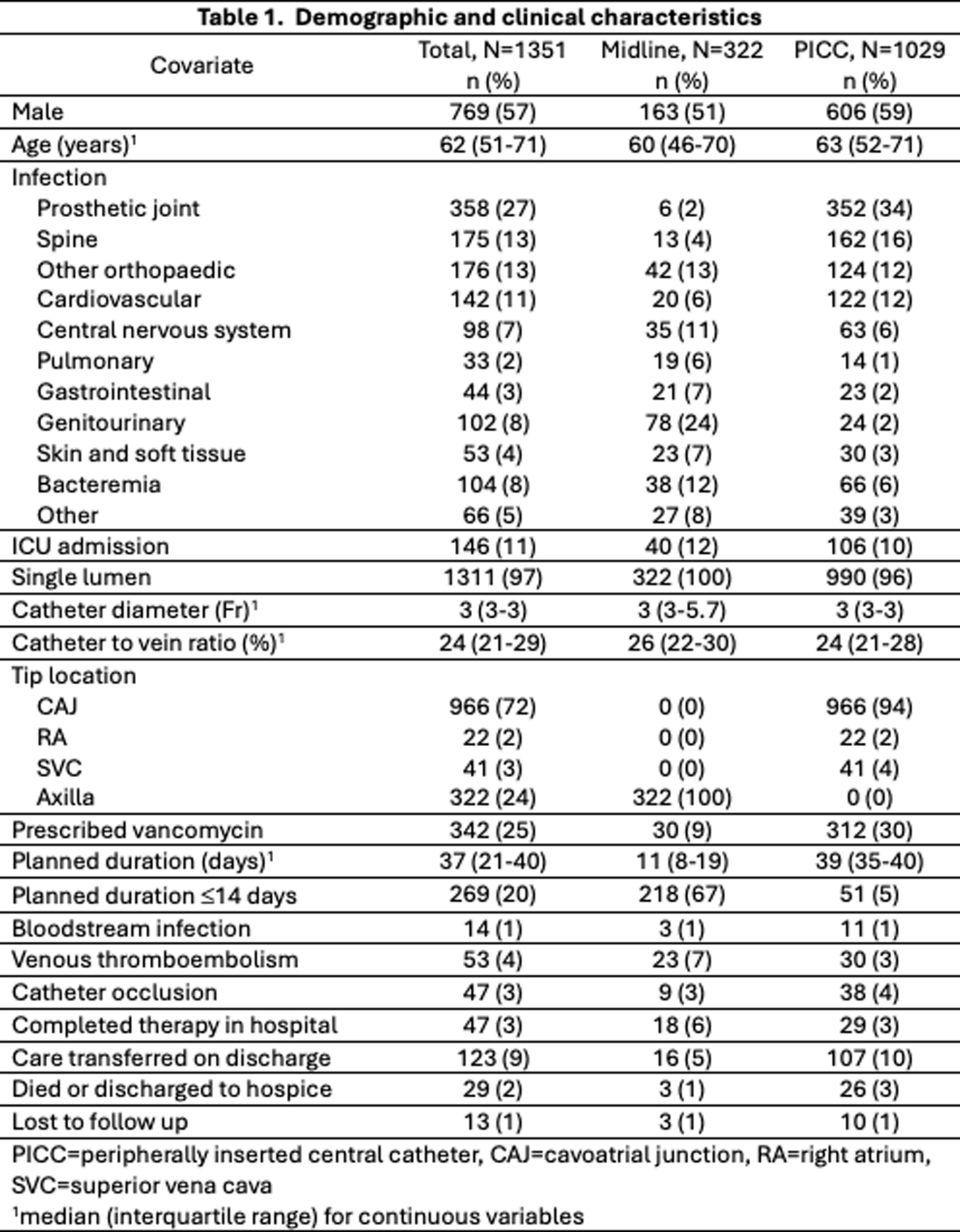

**Figure:**